# Two Cases of Adrenal Malignancy With Macroscopic Fat

**DOI:** 10.1210/jcemcr/luac029

**Published:** 2023-02-07

**Authors:** Tom Wilkinson, Penny Hunt, Alexandra McHaffie

**Affiliations:** Department of Endocrinology, Christchurch Hospital, Christchurch 8011, New Zealand; Department of Endocrinology, Christchurch Hospital, Christchurch 8011, New Zealand; Department of Medicine, University of Otago (Christchurch), Christchurch 8011, New Zealand; Department of Radiology, Christchurch Hospital, Christchurch 8011, New Zealand

**Keywords:** adrenocortical carcinoma, sarcoma, macroscopic fat

## Abstract

The presence of macroscopic fat on computed tomography (CT) imaging has been traditionally regarded as an indication that an adrenal lesion is likely to be a benign myelolipoma, for which further investigation is not usually required. Two cases are described where an adrenal lesion was eventually found to be malignant on histology (adrenocortical carcinoma in the first case, undifferentiated sarcoma in the second case), despite the presence of macroscopic fat on CT. In both cases there were other clinical and radiological indicators of potential malignant pathology. These cases add to increasing awareness in the literature that malignant adrenal tumors may rarely contain macroscopic fat, emphasizing a need for clinical vigilance.

Adrenal lesions are a common radiological finding. Although the vast majority are benign [1], some represent important malignancies. The presence of macroscopic fat on CT has been traditionally regarded as an indicator of benign pathology; however, placing too much diagnostic weight on one radiological marker may lead to misleading conclusions. Two recent cases at our institution illustrate this potential diagnostic pitfall, including an instance of a rare malignancy not previously described as manifesting macroscopic fat.

## Case 1

**Figure 1.**
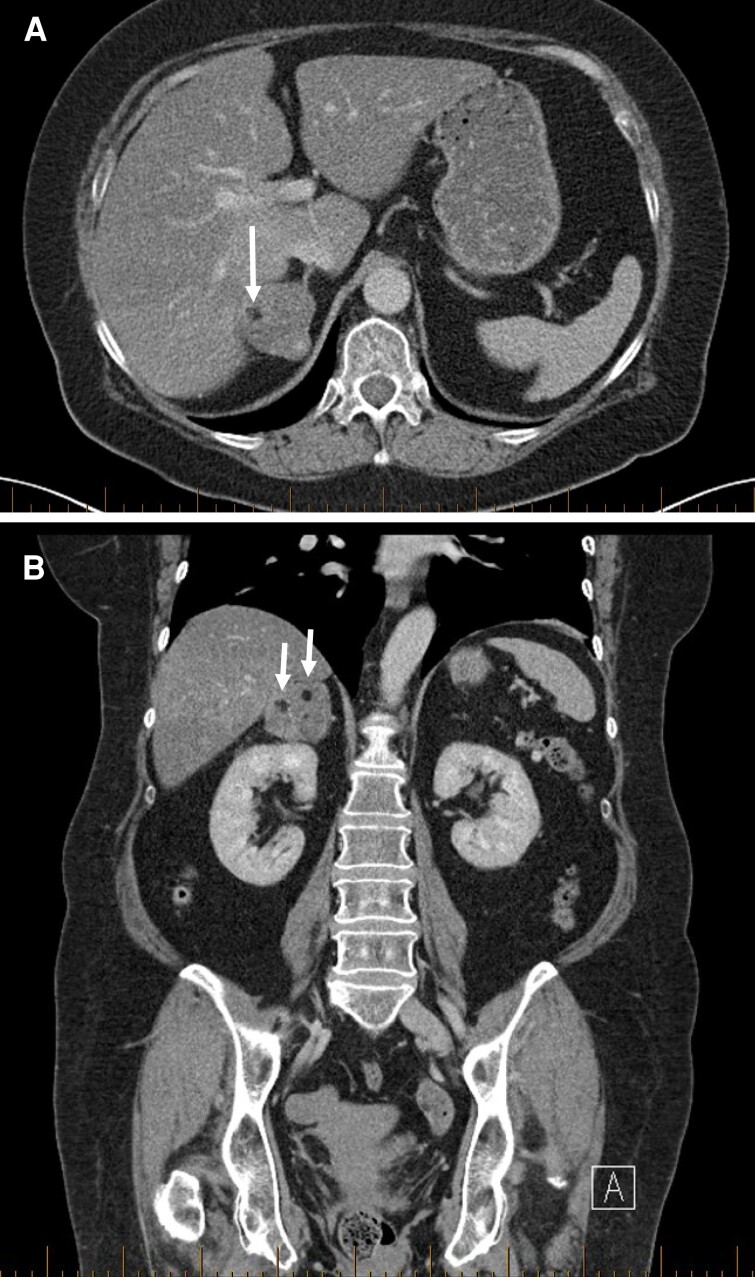
(A) Case 1, initial abdominal CT, axial portal venous phase. Focus of macroscopic fat indicated by arrow. (B) Case 1, initial abdominal CT, coronal portal venous phase. Two foci of macroscopic fat indicated by arrows.

A 62-year-old woman, initially presenting with abnormal liver function tests, was found to have a well-circumscribed lobulated heterogeneous mass in the right adrenal gland on CT abdomen measuring 4.9 cm in maximal dimension. Three hypodense foci, measuring up to 10 mm, were identified within the mass and thought to represent macroscopic fat on the basis of being visible to the eye and of similar density to fat seen elsewhere. The mass was therefore initially reported as consistent with adrenal myelolipoma. Later retrospective review measured density for 2 of these foci as −60 and −90 Hounsfield units, consistent with fat, and calculated this fat as comprising < 2% of the mass (using Vitrea software, Canon Medical Informatics, Inc.). Representative images are presented in Fig. 1. No prior imaging was available for comparison.

Results of functional testing included a significantly elevated 24-hour urine cortisol (3997 nmol [1445 mcg]; nr 100-400) and suppressed adrenocorticotropin hormone (ACTH) (< 0.2 pmol/L), consistent with adrenal-based Cushing syndrome. Plasma aldosterone was below the limit of quantification. 24-hour urine metanephrine, plasma testosterone, and plasma dehydroepiandrosterone sulfate (DHEA-S) were within the normal range.

Past medical history included hypertension and type 2 diabetes mellitus (possibly secondary to Cushing syndrome). There was no known history of any hematological disorder, nor any abnormality on initial full blood count.

CT abdomen also identified multiple hepatic lesions, unable to be characterized in part due to marked background hepatic steatosis. A subsequent hepatic magnetic resonance imaging (MRI) confirmed the presence of multiple lesions for which the differential diagnosis included metastases, adenomata, or multifocal hepatocellular carcinoma. The adrenal lesion was visualized on this MRI, with no change in size from the CT performed 26 days prior. The areas of interest within the lesion demonstrated high T1 and T2 signal with loss of signal on fat-suppression sequences and chemical shift artifact at their margins on opposed-phase imaging, consistent with macroscopic fat [2].

A hepatic core biopsy was obtained, with morphology and immunohistochemistry consistent with metastatic adrenocortical carcinoma. The patient proceeded to palliative treatment with mitotane (4.5 g daily in divided doses), chemotherapy (3 cycles of doxorubicin, etoposide, and cisplatin), and trans-arterial embolization of hepatic metastases. Cortisol excess required additional management with metyrapone (titrated to 4.5 g daily in divided doses) and ketoconazole (titrated to 1200 mg daily in divided doses). There was an initial response, with normalization of urine cortisol and stability of lesions on surveillance CT; however, the disease subsequently progressed and she died 14 months after the initial diagnosis was made.

## Case 2

**Figure 2.**
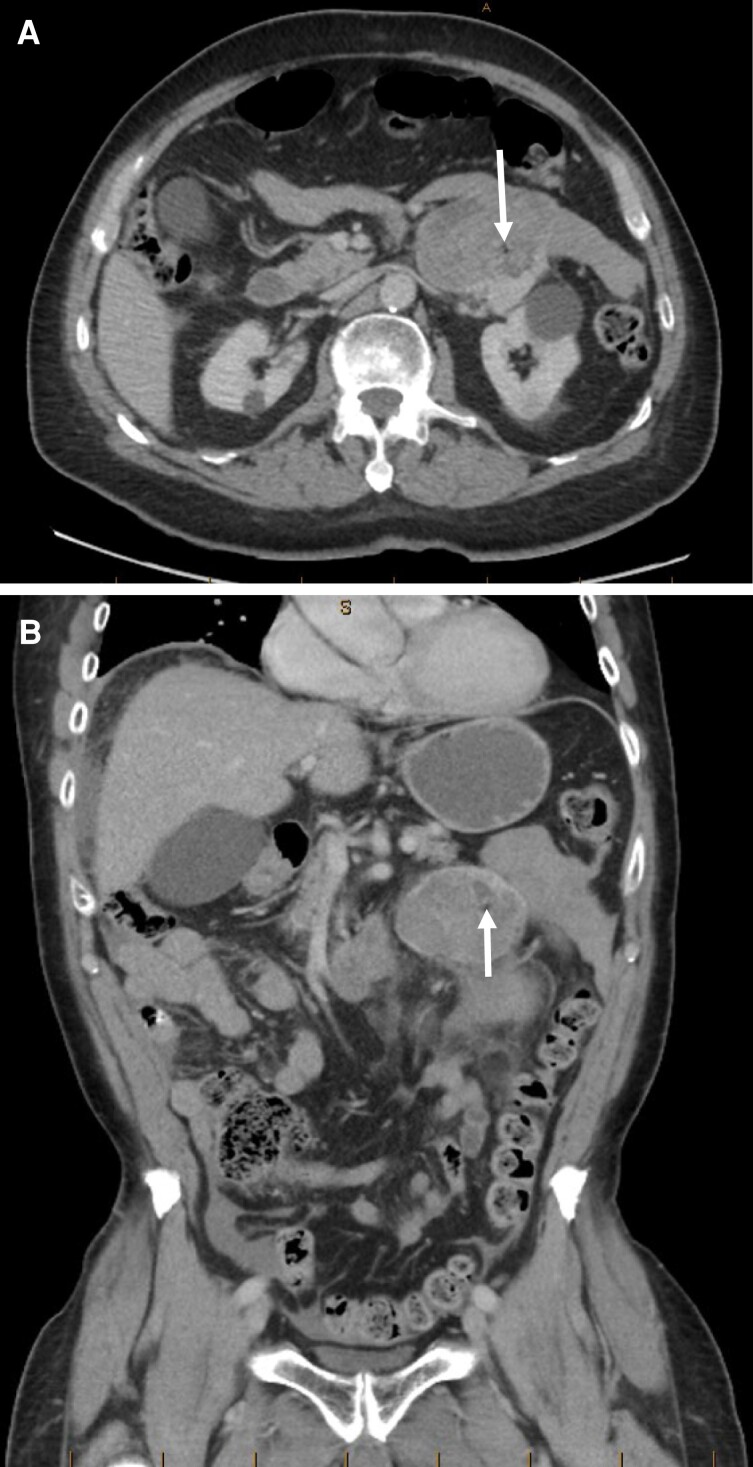
(A) Case 2, initial abdominal CT, axial portal venous phase. Focus of macroscopic fat indicated by arrow. (B) Case 2, initial abdominal CT, coronal portal venous phase. Focus of macroscopic fat indicated by arrow.

A 72-year-old man presented initially with abdominal pain and was found on CT to have a retroperitoneal lesion, contacting and possibly arising from the left adrenal gland. The lesion measured 7.6 cm in maximal dimension; however, there was associated acute retroperitoneal hemorrhage, limiting assessment of the true size and contours of the underlying mass. No prior imaging was available for comparison. A single hypodense focus was identified, measuring 7 mm and on later retrospective review calculated as comprising < 1% of the overall lesion. The focus was visible to the eye and of similar density to fat elsewhere (later retrospectively measured as −20 Hounsfield units) and was therefore thought to represent macroscopic fat. Representative images are presented in Fig. 2. Due to the presence of macroscopic fat the lesion was initially thought to be most compatible with a benign adrenal myelolipoma. Liposarcoma and adrenocortical carcinoma were acknowledged in the initial report as possible, but unlikely, diagnoses.

Plasma normetanephrine was elevated (2716 pmol/L [497 pg/mL]; nr < 900) in the context of acute illness. Cushing syndrome and primary aldosteronism were not formally excluded. Past medical history included hypertension controlled on candesartan, and hemochromatosis managed with regular blood donation. At presentation blood pressure was 127/75 mmHg and full blood count was normal.

The patient presented again 1 month later with ongoing abdominal pain. Repeat CT demonstrated a new small central hyperdense component to the lesion, suspicious for active extravasation. He proceeded to embolization of the left inferior adrenal artery. He was seen again 1 month after embolization, at which time a further CT showed the lesion to have enlarged to 9.0 cm.

The patient proceeded to open left adrenalectomy, left nephrectomy, and colonic resection. Histology was consistent with an undifferentiated spindling sarcoma, possibly representing a malignant peripheral nerve sheath tumor. Areas of tumor necrosis were noted. Intra-tumor macroscopic fat was not specifically identified.

Surveillance CT 4 months after surgery unfortunately showed widespread disease recurrence with extensive retroperitoneal and peritoneal metastases. The patient declined palliative chemotherapy and died 7 months after the mass was first identified.

## Discussion

The presence of macroscopic fat on imaging of an adrenal lesion has been traditionally thought to indicate benign myelolipoma. American College of Radiology recommendations for management of incidental adrenal masses regard this finding as essentially diagnostic for myelolipoma, with no additional workup or follow-up imaging generally required, although that recommendation was presented with a caveat that it should not be termed a formal “guideline” and that “Radiologists should feel comfortable deviating from the algorithm” [3].

A recent systematic review described 7 cases of adrenocortical carcinoma where macroscopic fat was seen on preoperative imaging [4]. Four cases had a small proportion of fat (< 5%); percentage fat was not reported for the remaining 3 cases. Three cases reported tumor size > 6 cm; size was not reported for the remaining 4 cases. The authors suggested consideration of follow-up imaging or biopsy as appropriate for patients with large (> 6 cm) symptomatic tumors with < 5% fat, although acknowledging significant uncertainty. Of note, our first case (maximal tumor dimension 4.9 cm) would not meet these criteria, while application to our second case would be problematic due to the presence of acute hemorrhage precluding calculation of the size of the underlying mass.

A 2009 pathologic case series of 3 patients with adrenocortical neoplasms, classified as either adrenocortical carcinoma or of uncertain malignant potential, described foci of myelolipomatous and lipomatous metaplasia [5]. This phenomenon may account for the radiologic finding of macroscopic fat in rare cases of adrenocortical carcinoma, particularly noting that in all such described cases (including our case) the proportion of fat was < 5% where quantified.

Although adrenocortical carcinoma has not been described with fat > 5%, the yield of using percentage fat to discriminate between myelolipoma and adrenocortical carcinoma is uncertain. Histologically, myelolipoma comprises a mixture of mature fat and hematopoietic cells, however the relative proportion of these elements can vary significantly. Consequently, radiological appearances range from tumors that appear to almost entirely consist of macroscopic fat to tumors where visible fat is nonexistent [6].

In both cases described here, macroscopic fat comprised a very small component of the lesion, raising a question of whether, in retrospect, the possibility of malignancy could have been given greater weight at the outset. However, the rarity of adrenocortical carcinoma with macroscopic fat, compared to the well-described phenomenon of myelolipoma containing only a small proportion of fat, suggests that myelolipoma remains the more likely diagnosis even where fat is < 5%. Radiologists must weigh providing an exhaustive differential diagnosis, including very rare entities, with the potential risks of this approach, including harm and costs generated by overinvestigation. Of note, the initial radiologist report for case 2 suggested malignancy in the differential diagnosis.

Adrenal tumor size can be used as an indication of the likelihood of malignancy; however, it is unlikely to be a reliable discriminator when specifically differentiating adrenocortical carcinoma and myelolipoma. One study, using data from patients undergoing adrenalectomy, estimated the population rate of malignancy as 10% in tumors ≥ 4 cm, 19% in tumors ≥ 6 cm, and 47% in tumors ≥ 8 cm [7]. This is in contrast to a review estimating the average size of myelolipomata described in the literature as 10.2 cm [8]. Consequently, recommendations to consider adrenalectomy for tumors ≥ 4 cm cannot be readily applied where myelolipoma is thought likely [2].

Our second case was found to have a poorly differentiated sarcoma. We are not aware of previous descriptions in the literature of this manifesting as an apparent adrenal tumor with macroscopic fat. Even if the reporting radiologist had provided an exhaustive differential diagnosis for the initial CT, it would almost certainly have not contained the final diagnosis in this case.

These factors collectively suggest caution in using isolated radiology findings (macroscopic fat, tumor size) to classify an adrenal tumor as likely benign, without factoring in the broader clinical context. Both cases described had elements to their presentation raising suspicion of malignancy.

Case 1 had evidence of endocrine functionality. This is well-described in adrenocortical carcinoma, with 62% reported to be functional in one review, of which Cushing syndrome was the most common abnormality [9]. Of note, where an adrenocortical neoplasm contains myelolipomatous metaplasia, it is thought that the myelolipomatous tissue can promote cortical or medullary hyperfunction [5]. Adrenal myelipomata, which comprise adipocytes and hematopoietic cells, are less likely to be associated with endocrine abnormalities, although adrenal hormonal production has been described in 7.5% of cases, while an additional 10% of cases occur in the setting of congenital adrenal hyperplasia, possibly as a result of ACTH excess [8].

Of more significance, both of our patients had clinical suspicion of malignancy prior to histological diagnosis, with hepatic lesions suspicious for metastases in case 1 and possible growth of the mass in case 2. Although frequently large in size, adrenal myelolipomata are typically slow growing. One series of 69 patients with radiographically diagnosed myelolipoma described growth in 11, at a median rate of 0.16 cm/year [10]. In contrast, the mass was seen to change in size by 1.4 cm over 2 months in our second case, although interpreting this finding is complicated by hemorrhage.

It is important to note that histology did not identify macroscopic fat in case 2, and that macroscopic fat was unable to be histologically confirmed in case 1 (adrenalectomy was not clinically indicated). There were, however, multiple radiological features present that were consistent with macroscopic fat. Although tumor necrosis or cystic change may result in hypodense foci, radiologically these would be expected to manifest as fluid density rather than fat density. Ultimately, the decision to proceed to adrenalectomy due to suspicion of malignancy rests on clinical and radiological factors, rather than on histological findings which are only known once adrenalectomy has occurred.

## Learning Points

- The radiological finding of macroscopic fat in an adrenal lesion is highly suggestive of benign myelolipoma; however, it does not completely exclude malignancy, particularly when the proportion of fat is < 5%.
- Tumor size is not useful in discriminating between adrenal myelolipoma and adrenocortical carcinoma; however, rapidly growing lesions are more likely to be malignant.
- Ongoing vigilance is important when a clinical presentation has suspicious features for malignancy, even if isolated radiology findings are seemingly reassuring.

## Data Availability

Data sharing is not applicable to this article as no datasets were generated or analyzed during the current study.

## Abbreviations

ACTH: adrenocorticotropic hormone
CT: computed tomography
